# Preliminary Evidence for Global Properties in Human Listeners During Natural Auditory Scene Perception

**DOI:** 10.1162/opmi_a_00131

**Published:** 2024-03-26

**Authors:** Margaret A. McMullin, Rohit Kumar, Nathan C. Higgins, Brian Gygi, Mounya Elhilali, Joel S. Snyder

**Affiliations:** Department of Psychology, University of Nevada, Las Vegas, Las Vegas, NV, USA; Department of Electrical and Computer Engineering, Johns Hopkins University, Baltimore, MD, USA; Department of Communication Sciences & Disorders, University of South Florida, Tampa, FL, USA; East Bay Institute for Research and Education, Martinez, CA, USA

**Keywords:** auditory scene perception, acoustic analysis, natural scenes, computational modeling

## Abstract

Theories of auditory and visual scene analysis suggest the perception of scenes relies on the identification and segregation of objects within it, resembling a detail-oriented processing style. However, a more global process may occur while analyzing scenes, which has been evidenced in the visual domain. It is our understanding that a similar line of research has not been explored in the auditory domain; therefore, we evaluated the contributions of high-level global and low-level acoustic information to auditory scene perception. An additional aim was to increase the field’s ecological validity by using and making available a new collection of high-quality auditory scenes. Participants rated scenes on 8 global properties (e.g., open vs. enclosed) and an acoustic analysis evaluated which low-level features predicted the ratings. We submitted the acoustic measures and average ratings of the global properties to separate exploratory factor analyses (EFAs). The EFA of the acoustic measures revealed a seven-factor structure explaining 57% of the variance in the data, while the EFA of the global property measures revealed a two-factor structure explaining 64% of the variance in the data. Regression analyses revealed each global property was predicted by at least one acoustic variable (R^2^ = 0.33–0.87). These findings were extended using deep neural network models where we examined correlations between human ratings of global properties and deep embeddings of two computational models: an object-based model and a scene-based model. The results support that participants’ ratings are more strongly explained by a global analysis of the scene setting, though the relationship between scene perception and auditory perception is multifaceted, with differing correlation patterns evident between the two models. Taken together, our results provide evidence for the ability to perceive auditory scenes from a global perspective. Some of the acoustic measures predicted ratings of global scene perception, suggesting representations of auditory objects may be transformed through many stages of processing in the ventral auditory stream, similar to what has been proposed in the ventral visual stream. These findings and the open availability of our scene collection will make future studies on perception, attention, and memory for natural auditory scenes possible.

## INTRODUCTION

Every day, our auditory system undertakes the complex task of organizing various incoming sounds in a coherent manner, allowing us to not only decipher where sounds are coming from, but to also interpret what we are listening to. For example, when conversing with a friend at a noisy café, the auditory system maintains the exceptional ability to segregate the noisy background (e.g., music, espresso machines, other conversations) from a friend’s voice and further group the sound components of their speech into an intelligible stream of words. The process of perceptually segregating and grouping numerous acoustic objects is known as ‘Auditory Scene Analysis’ (ASA; Bregman, 1990).

Historically, theories of both auditory and visual scene analysis have suggested that our perception of a scene relies on the identification and segregation of multiple objects within it, resembling a detail-oriented processing style (Biederman, 1987; Bregman, 1990). However, it is possible that a more global process may also occur when observers evaluate auditory scenes. In the visual modality, there is evidence for global properties that enable visual scenes to be rapidly recognized, even without recognition of individual objects comprising the scene (Greene & Oliva, 2009a, 2009b; Ross & Oliva, 2010). The significance of the scene-centered approach proposed by Greene and Oliva (2009a) is that the representation exists at the level of the entire scene and not just individual objects. Instead of just building a visual representation using local geometric information about individual objects, the visual system also uses global properties that provide information about the scene’s structure, function, and overall layout to guide perception. The global properties identified by Oliva and colleagues fall into three categories: structural properties (openness, expansion, mean depth), constancy properties (temperature, transience), and functional properties (concealment, navigability). Greene and Oliva (2009b) asked participants to view a series of visual scenes and indicate whether each scene was consistent with a basic-level category (e.g., identifying a scene as a mountain or waterfall) or a global category (e.g., identifying a scene as an open environment or hot place). The authors controlled the presentation rate of each image to maintain participant classification accuracy at 75%. The results indicated participants required less viewing time to perform the global categorization task at the same level of accuracy as the basic-level task. Other studies have demonstrated the importance of global image features (Greene & Oliva, 2009a; Oliva & Torralba, 2006; Ross & Oliva, 2010) and collectively suggest that these global properties could act as automatic heuristics to analyze natural visual scenes.

Recent work has also highlighted the importance of individual objects in scene categorization. Wiesmann and Võ (2022) asked participants to rapidly categorize visual scenes with various levels of spatial resolutions (ranging from low to high) into a superordinate category (indoor or outdoor) and also a basic-level category (e.g., kitchen, living room, beach, forest, etc.). Participants were able to perform this task well above chance, but performance was higher for scenes with higher spatial resolutions. Furthermore, participants were even more accurate at identifying individual objects in scenes with higher resolutions, suggesting rapid scene categorization cannot be explained by global properties alone, but likely also relies on information provided by individual objects. This finding was extended by Wiesmann and Võ (2023), who showed that participants were able to successfully identify scenes that were reduced to a single object (e.g., TV, car, bed, etc.) at speeds less than 50 msec. These findings demonstrate the complexity of visual scene processing and consider the roles played by the setting of scenes as well as the individual objects within them. Our primary research goal was to evaluate the contribution of high-level semantic knowledge and low-level acoustic information during auditory scene perception. As previously mentioned, many studies have demonstrated the usefulness of global information in rapid scene categorization, but more recent endeavors suggest individual objects also play a crucial role in scene categorization. In the auditory domain, the role of high-level semantic information and low-level acoustic features has been explored in the change deafness literature. Characterized as a perceptual error, change deafness is the inability to detect changes in auditory scenes (Snyder et al., 2012). This error is useful in our study of ASA because it informs us of our auditory system’s limitations. To study change deafness, participants are typically presented with a simultaneous array of sounds (e.g., dog barking, piano, phone ringing, bell). After a short interruption (usually white noise), the scene is presented again. Finally, participants must indicate whether the second scene was the same as or different than the first scene. In one such study, Gregg and Samuel (2009) presented participants with two types of different trials (i.e., the scene changed in some way from the first to second presentation). These trials exhibited either a within-category change (e.g., a small dog bark changing to a large dog bark) or a between-category change (e.g., a small dog bark changing to a bell chime). Results demonstrated that participants’ ability to detect a within-category change was significantly worse than their ability to detect a between-category change. As the low-level acoustic features were controlled for, the authors hypothesized that semantic information is useful when constructing representations of auditory scenes. They further observed that listeners used both high-level semantic information and low-level acoustic information when constructing auditory representations of sounds, but the low-level acoustic information was not used to the same extent as semantic knowledge of sounds.

Additional research has also evaluated the influence of acoustic features of sounds on listeners’ ability to identify and discriminate recognizable objects, both in isolation and when presented concurrently with complex auditory scenes. Leech et al. (2009) addressed the possible existence of semantic knowledge-driven expectancies about auditory scenes. In their study, participants were presented with multiple target sounds embedded into an auditory scene. The target sounds were either congruent (e.g., the target being a goat and the background being a farm) or incongruent (e.g., the target being a goat and the background being a casino) with the auditory scene in which the sounds were embedded. An acoustic analysis of all target sounds and background auditory scenes was conducted to evaluate whether acoustic similarity or dissimilarity between the targets and backgrounds may have influenced target identification. Participants more accurately identified target sounds that were contextually incongruent with the background scenes and the acoustic variables that significantly influenced this effect were correlogram-based pitch measures and peak autocorrelation statistics. However, since acoustic similarity was not an exclusive predictor of target congruency or incongruency with the background scene, the findings from this study suggest that high-level semantic factors may significantly influence listeners’ ability to detect and identify meaningful sounds within complex auditory scenes.

The tasks just described are useful for the study of ASA. However, they typically use a mixture of simultaneously presented sounds from different recordings (Gregg & Samuel, 2008, 2009; Leech et al., 2009). This presents a fundamental limitation to studies of this type: the stimuli are somewhat artificial in nature, especially since some of the sound combinations may not typically occur in the real-world. An example of a study using more naturalistic sounds is by McDermott and Simoncelli (2011), which examined sound texture perception using a computational model of the human auditory system. Sound textures, which are the result of numerous similar acoustic events occurring in succession (e.g., rainstorm, galloping horses), were processed using an auditory model based on the tuning properties of neurons from the cochlea to the thalamus. To better understand how sound textures are represented in the brain, the authors then synthesized the sound textures based on the output of their model (i.e., the statistics of real-world sounds). They hypothesized that if the novel synthesized sound textures were statistically matched with those of the real-world sounds, then the brain should be able to achieve texture recognition due to the synthesized signal sounding like a version of the originally presented sound texture. In a series of behavioral experiments, they found that synthetic sound textures were recognizable to participants but eliminating some of the statistics in the model reduced performance. Additionally, the authors modified the model so that it was less representative of the mammalian auditory system, which resulted in reduced recognizability of the synthetic sound textures. Some of the synthesized sounds (e.g., wind chimes, tapping rhythm, and a person speaking English) were not recognizable, though. These findings suggest that sound texture perception arises from the recognition of simple statistics in early auditory representations, which are potentially computed in neural populations downstream from the peripheral auditory system. Ultimately, the results of this study are important to our understanding of how the auditory systems analyzes naturalistic sounds and could inform us of how the auditory system processes more complex stimuli, like naturalistic auditory scenes. By using naturalistic auditory scene stimuli, we hope to further increase the range of ecologically valid stimuli and abilities under study in the field of auditory perception.

Our secondary research goal was to record and use real-world auditory scenes. One major limitation in the current body of literature is the consistent use of pure tones, noise bursts, or artificially contrived auditory scenes as stimuli to study ASA. While using such stimuli has revealed much about the fundamental mechanisms of ASA and auditory perceptual awareness, the findings resulting from this work have limited power in educating us about natural auditory scene processing. In the field of visual scene perception, the use of naturalistic stimuli is highly evident (Greene & Oliva, 2009a, 2009b, 2010; Hansen et al., 2018; Harel et al., 2016; Ross & Oliva, 2010). This is perhaps the case because there are numerous large databases of natural visual scenes openly available for public use (Geisler & Perry, 2011; Xiao et al., 2010). While there are some databases that include high quality clips of individual sound objects (e.g., a single dog bark; Gygi & Shafiro, 2010), no database of high quality, real-world auditory scenes currently exists to our knowledge. To address this issue, we recorded a relatively large volume of audio/visual scenes, which are available for other researchers to use (see Methods for the database link).

By embracing the complexity of natural auditory scenes, a third goal of this study was to explore the relationship between complex auditory scenes and global perception through the lens of deep neural network models. In many ways, these artificial models parallel biological processing of sensory information, particularly in their ability to provide rich mappings from low-level features to more complex decompositions of sensory inputs (Richards et al., 2019; Saxe et al., 2021). Hierarchical feature analysis and abstraction is a hallmark of both deep neural networks and sensory systems, particularly in the ventral stream. This approach has been embraced in vision studies which showed that deep neural networks trained on identifying objects reveal progressions of hierarchical processing from simple features like edges and textures to more complex object categories mimicking gradients of processing in the visual what stream (Güçlü & van Gerven, 2015). Similar insights about processing gradients in the auditory stream have been gleaned using deep learning methods that not only shed light on cortical processing hierarchies, but also specialized processing in non-primary cortical pathways for processing speech and music sounds (Kell et al., 2018; Wang et al., 2022). In the current study, deep neural networks were also used as an investigative tool to infer relationships between behavioral responses to natural auditory scenes and nonlinear abstractions of these scenes extracted by learned models. This approach extends the correlations inferred from low-level acoustic features and sheds light on the balance between recognizing discrete sound events and perceiving global characteristics of a natural scene.

### Purpose of Present Study

The present study aimed to evaluate the contributions of high-level global properties and low-level acoustic features to natural auditory scene perception. Participants listened to 200 auditory scenes and made a series of global property judgments on them. Additionally, we conducted an acoustic analysis on all 200 scenes with the goal of understanding how these features are related to global processing of auditory scenes. We predicted there would be a general consistency on all eight global property ratings of each auditory scene across participants, which was measured using intraclass correlations of each rating scale. We conducted two separate factor analyses on the average global property ratings and acoustic measures of each scene to determine the number of factors that characterize the variability found within scene judgments and within the array of acoustic features. An additional eight multiple linear regression analyses were also conducted to predict performance on the global property rating task based on the acoustic features of the scenes. We did not originally plan on conducting this analysis; however, we decided it was more helpful for directly testing the relationships between the average global property ratings and acoustic measures of each scene.

Finally, we deploy two state-of-the-art deep neural networks, both carefully tailored to map the intricate acoustic signals onto high-dimensional embeddings. The first model is optimized to pinpoint specific sound events within a scene—whether it be the chirping of a bird, the chatter of human voices, or the distant bark of a dog. The second model is trained to identify the general setting of a scene, be it a bustling kitchen or a quiet office. This analysis aims to address a central question in auditory perception: Is the broader context of an auditory scene captured first, subsequently aiding in the recognition of specific sound events (global-to-local processing)? Or do we first detect individual sounds and then integrate them to make sense of the scene as a whole (local-to-global processing)?

## METHODS

### Ethics Statement

All procedures were approved by the University of Nevada, Las Vegas (UNLV) Institutional Review Board. All de-identified experimental data, analysis techniques, protocols, and stimuli are available on this project’s Open Science Framework repository (https://osf.io/zj4xe/).

### Auditory Scene Collection and Database

We recorded and processed 200 auditory scenes from various locations across the United States we believed to represent typical environments humans are exposed to, such as parks, classrooms, hiking trails, city streets, forests, and cafes. There are multiple recordings in similar but distinct settings (e.g., different cafes, parks, etc.). The scenes differ in the number of sound sources, but each scene contains more than one source (e.g., talking, wind, a bird chirping). Using a standardized recording procedure, we placed a Zoom Q8 camcorder (Zoom North America, Inc., Hauppauge, NY) on a tripod and made one-minute recordings at each location, noting various aspects of the scene, such as the date, time of day, cardinal direction the camcorder was pointing, temperature (°F), sounds observed, and any additional notes about the recording. After each recording session, the field notes were digitized into a spreadsheet for ease of organization and file identification. We then listened to each recording and confirmed all sounds identified in the field notes were heard in the recording. Next, we edited each minute-long recording into a four-second-long version which best characterized the scene location and included more than one sound object. Our collection of auditory scenes, including detailed descriptions of each scene can be found on our project’s OSF repository.

### Participants

68 English-speaking adults (48 female) aged 18–25 (mean = 21.19 years) with no known hearing, visual, or neurological deficits were recruited from the UNLV participant pool and across the United States. Participants from the participant pool were reimbursed with course credit and participants external to the university volunteered for no compensation. In total, 142 participants were excluded from this study because they did not speak English, did not complete the experiment, did not have normal hearing, had any type of severe neurological or psychiatric disorder (e.g., schizophrenia, bipolar disorder, stroke, traumatic brain injury), or failed the headphone check, compliance check, and/or attention check (see below for criteria and descriptions of tasks).

### Stimuli

We selected a total of 200 auditory scenes for this experiment based on common heuristics for exploratory factor analysis sample sizes (Pearson & Mundform, 2010). Stimuli consisted of 200 naturalistic auditory scenes originating from our database of acoustic scenes. Each scene was four seconds in length and matched for RMS amplitude. A linear on-ramp from zero amplitude was imposed on the first and last 10 msec of each sound clip to avoid introducing artifacts due to abrupt sound onsets and offsets (Gregg et al., 2014, 2017; Gregg & Samuel, 2008, 2009).

### Procedure

Participants were provided a link to complete the experiment online via Qualtrics (Qualtrics, Provo, UT). Experiment links can be found on our project’s OSF repository. Informed consent was obtained online from each participant before they began the experiment. Participants were asked to complete the study on a desktop or laptop computer using headphones and while in a quiet environment. In total, the experiment took 60–120 minutes to complete. The experiment consisted of four sections: 1) a headphone check, 2) the global property rating task, 3) a compliance and attention check, and 4) a demographic questionnaire.

#### Headphone Check.

Because this was an online study of auditory perception, we tested each participant’s sound quality by administering a headphone check. This test consists of 6 trials of a 3-AFC intensity discrimination task (Woods et al., 2017). Participants were asked to indicate which tone had the lowest volume by selecting one of three button options labeled “Tone 1”, “Tone 2”, or “Tone 3.” Any participants who did not correctly answer five out of the six trials were excluded from the study.

#### Global Property Rating Task.

**Figure 1.**
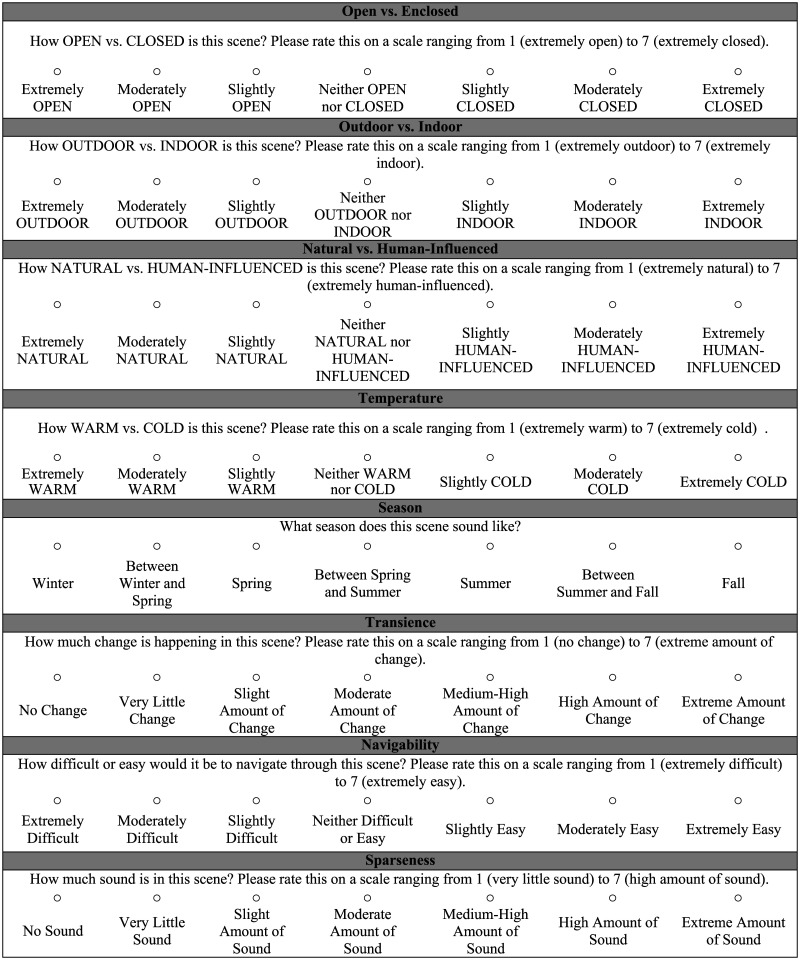
Global Property Rating Scales.

Each participant was asked to judge all 200 scenes on four different global properties on a Likert scale ranging from 1 (lowest extreme) to 7 (highest extreme (see Figure 1 for full descriptions of each rating scale provided to participants). The global properties chosen for this experiment were based on the work of Greene, Oliva, and colleagues (Greene & Oliva, 2009a, 2009b; Oliva & Torralba, 2001, 2006) and were properties we thought might be perceived in the auditory domain. Although we were unsure if participants would be able to hear some of the properties, such as season and temperature, we wanted to have a good number of properties whose ratings could be submitted into the factor analysis. Participants were allowed to listen to each scene as many times as they needed to make each of the four judgments. The eight global property judgments were pseudo-randomized across participants using a Latin square design, with each global judgment type only appearing in each possible position once (see Table 1 for order of questions in each condition). Participants were randomly assigned to one of eight condition groups. Groups 1–4 (n = 32) made the following global property judgments on each scene: Open vs. Enclosed, Outdoor vs. Indoor, Natural vs. Human-Influenced, and Temperature, and Groups 5–8 (n = 36) made judgments on each scene’s Season, Transience, Navigability, and Sparseness.

**Table 1.** The order of rating scales questions in each condition.

Group	Order of Rating Scale Questions			
1	Open vs. Enclosed	Outdoor vs. Indoor	Natural vs. HI	Temperature
2	Outdoor vs. Indoor	Open vs. Enclosed	Temperature	Natural vs. HI
3	Natural vs. HI	Temperature	Open vs. Enclosed	Outdoor vs. Indoor
4	Temperature	Natural vs. HI	Outdoor vs. Indoor	Open vs. Enclosed
5	Season	Transience	Navigability	Sparseness
6	Transience	Season	Sparseness	Navigability
7	Navigability	Sparseness	Season	Transience
8	Sparseness	Navigability	Transience	Season

*Note*. HI = Human-Influenced.

#### Compliance and Attention Check.

We used a set of questions from Mehr et al. (2018) to ensure participants were adequately attending to the experimental task. The following question was dispersed throughout the global property rating task: 1) “What color is the sky? Please answer this incorrectly, on purpose, by choosing RED instead of blue.”, with the response options of “Green,” “Red,” “Blue,” or “Yellow.” The correct response option (“Red”) was changed upon each presentation (e.g., the correct response was only presented in each answer slot once). Any participant who did not select this response option was excluded.

Upon completion of the rating task, participants were asked the following compliance questions:

1)  “People are working on this task in many different places. Please tell us about the place you worked on this task. Please answer honestly.” The response options for this question were: “I worked on this study in a very noisy place, I worked on this study in a somewhat noisy place, I worked on this study in a somewhat quiet place, or I worked on this study in a very quiet place.” Any participant who answered with “I worked on this study in a very noisy place” or “I worked on this study in a somewhat noisy place” was excluded.
2)  “Please tell us if you had difficulty loading the sounds. Please answer honestly.” The response options for this question were “Yes” or “No.” Any participant who responded with “Yes” was excluded.
3)  “How carefully did you complete this experiment? Please answer honestly. The response options for this question were: “Not at all carefully,” “Slightly carefully,” “Moderately carefully,” “Quite carefully,” or “Very carefully.” Any participant who answered with “Not at all carefully,” “Slightly carefully,” or “Moderately carefully” were excluded.

#### Demographic Questionnaire.

Lastly, participants completed a demographics questionnaire which asked about their health history and engagement with music. Additional questions were asked about participants’ auditory environment (e.g., time spent in various environments). Data collected from this questionnaire has not been included in the analyses reported here.

### Acoustic Analysis

To quantitatively gauge how low-level information may be used to understand auditory scenes, an extensive acoustic analysis was conducted on all 200 auditory scenes. The acoustic analyses chosen for this study have been used in various prior studies (Ballas, 1993; Gygi et al., 2007; Houtgast & Steeneken, 1985; Leech et al., 2009; Slaney, 1995) and were executed in MATLAB (MATLAB version 9.10 (R2021a)). A description of each acoustic measure is listed below.

#### Envelope-based Intensity and Rhythm Measures.

(1) Long-term RMS/Pause corrected RMS, which indicates the amount of silence present within each auditory scene; (2) number of peaks, where a peak is defined as a point in the vector that has a greater amplitude than the previous point by at least 80% of the range of amplitudes present in the vector; (3) number of bursts, showing an increase in amplitude of at least 4 dB lasting at least 20 msec (Ballas, 1993); (4) total duration; and (5) burst duration/total duration, a measure of how “rough” the envelope is.

#### Autocorrelation Pitch Statistics.

(1) Number of peaks; (2) maximum peak; and (3) standard deviation (SD) of the peaks. In this autocorrelation function, the peaks express periodicities in the waveform. The distribution of periodicities across various frequencies as well as the strength of a periodicity are evaluated in this measure.

#### Correlogram-based Pitch Measures.

(1) mean pitch; (2) median pitch; (3) SD pitch; (4) maximum pitch; (5) mean pitch salience; and (6) maximum pitch salience. This measure evaluates pitch and pitch salience by autocorrelating in sliding 16 msec time windows.

#### Moments of the Spectrum.

(1) mean; (2) SD; (3) skew; and (4) kurtosis. This measures the distribution of energy related to the overall timbre of the scene.

#### RMS Energy in Octave-wide Frequency Bands.

Gygi et al. (2007) used frequency bands ranging from 63–16,000 Hz. This measures the distribution of energy separately for different frequencies.

#### Spectral Shift in Time Measures.

(1) Centroid mean; (2) centroid SD; (3) mean; (4) SD; and (5) maximum centroid velocity. The measures of the centroid mean and SD are established on sequential 50-msec time windows throughout the entirety of the waveform, while the measure of spectral centroid velocity is determined by calculating the overall change in the spectral centroid across sliding 50-msec time widows.

#### Modulation Spectrum Statistics.

Proposed by Houtgast and Steeneken (1985), the modulation spectrum displays intermittent temporal variations in the envelope of a scene. This measure “divides the signal into frequency bands approximately one critical band wide, extracts the envelope in each band, filters the envelope with low-frequency bandpass filters (upper *f*_o_ ranging from 1–32 Hz), and determines the power at that frequency. The result is a plot of the depth of modulation-by-modulation frequency. The statistics measured here will be the height and frequency of the maximum point in the modulation spectrum, as well as the number, mean, and variance of bursts in the modulation spectrum (using the burst algorithm described above; Gygi et al., 2007, p. 846).

#### Spectral Flux Statistics.

Spectral flux evaluates how much change occurs in the spectrum at various frequency bands. This measure can potentially show salient changes in energy, which can be deduced as moments in time where a change in energy may capture the observer’s attention, further influencing their perception of the scene.

### Computational Models

#### Neural Network Models.

Two computational models are tested to infer relationships between human judgments of global properties and processing granularity of the scenes: An event-based model which emphasizes a more local analysis of sound events in a scene versus a setting-based model which emphasizes a more global analysis of the setting of the scene. In the first model, we employ a state-of-the-art event-based deep neural network, hereafter referred to as *M*_*event*_, based on the YAMnet architecture (Howard et al., 2017). The model was trained on the Audioset-YouTube database (Gemmeke et al., 2017), consisting of 2 million diverse audio samples taken from YouTube and containing a wide range of soundscapes spanning the broad classes of “Human sound,” “Animal sounds,” “Natural sounds,” “Music,” “Sounds of things,” “Source-ambiguous sounds,” and “Channel, environment, and background.” *M*_*event*_ was trained to recognize 521 unique audio event classes. The model is a convolutional neural network consisting of a first 1D convolutional layer, followed by 13 layers of depth-wise separable convolutions then two fully connected linear layers. The computational complexity as well as processing time constants increase as the input signal is analyzed through the different model layers, akin to the increased complexities and tuning observed along the hierarchy in sensory biological systems (Sharpee et al., 2011). For the current study, we focused on the first 14 processing layers of the model, excluding the final two fully connected layers. The 200 scenes used in the behavioral study were analyzed through the event-based model by first resampling each scene waveform at 16 kHz and normalizing within the range of [−1.0, +1.0]. Each waveform is then mapped using a short-time Fourier transform with a 25 msec Hann window and 10 msec hop, then a 64-bin mel-spectrogram spanning 125–7500 Hz was computed. Finally, the input was segmented into 1-second segments, each sampled into 96 frames, to match the trained architecture of YAMnet. Latent representations at each layer of the model were then concatenated across time segments to reconstruct the mapping over the entire scene duration. The embeddings at each layer were then integrated over time for each scene and yielded a matrix [*K*_*e*_, *F*_*e*_]^*l*^, where *K*_*e*_ represents the number of kernels in each layer, *F*_*e*_ represents frequency and *l* is the layer index.

**Figure 2.**
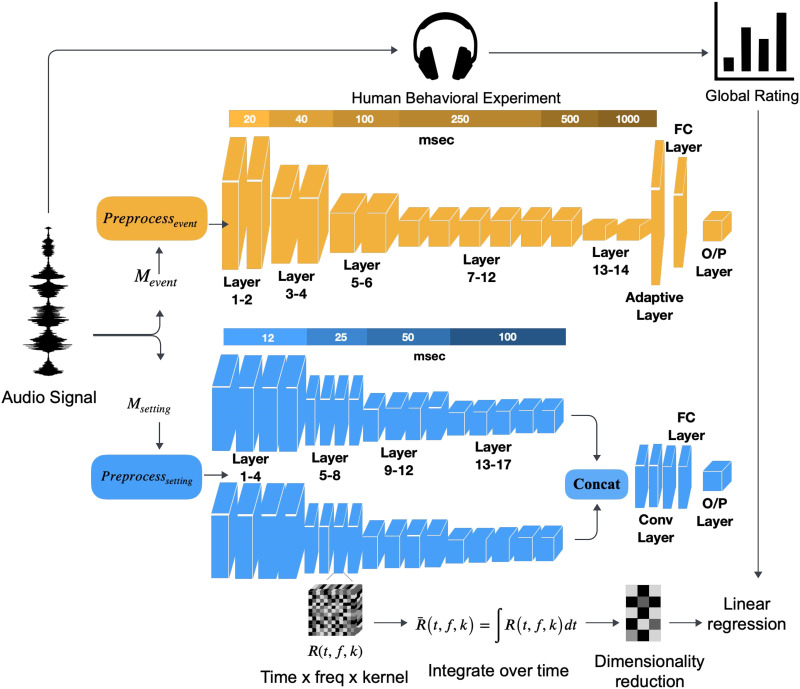
*Note*. A scene used in the behavioral study is given as input to both the event-based (*M*_*event*_) model (top, yellow) and setting-based model (*M*_*setting*_) model (bottom, Blue). The scene is analyzed through both models yielding abstract embeddings along different layers of each model. The representations from each layer and each model are extracted following the schematic in the bottom of the figure. High-dimensional tensors along Time × Frequency × Kernel are first integrated over time, then reduced in dimensionality. A linear regression is then performed to contrast the relationship between model embeddings and human judgments of global scene properties. The time constants (in msec) above each model reflect the different sampling rates at which the convolutional processes operate for each layer in each model.

In parallel, the same scenes were analyzed through a separate, state-of-the-art setting-based model. This model, hereafter referred to as *M*_*setting*_, consisted of a Resnet-like architecture (Hu et al., 2020) and was trained to classify different acoustic scenes. The model was trained on the TAU Urban Acoustic Scenes 2020 Mobile dataset (Heittola et al., 2020) to identify 10 unique scene settings: “airport,” “shopping mall,” “street pedestrian,” “metro station,” “public square,” “street traffic,” “tram,” “bus,” “metro,” and “urban park.” Each of the 200 scenes used in the behavioral study were analyzed through this *M*_*setting*_ model by resampling each scene at 44.1 kHz then mapping the time waveform using a 2048 Fast Fourier Transform (FFT) point process with 46 msec window and 23 msec hop interval. Next, a 128 log Mel filter bank was derived augmented with log Mel delta and delta-delta features (Rabiner & Schafer, 2010). Each input tensor was normalized along the frequency dimension within the range [0,1]. The model analyzes the input spectrogram using two paths focusing on different spans of the frequency axis, with the first path focusing on the lower frequency Mel bins (0–63) and the second path on the higher frequency Mel bins (64–127). These partial spectrograms are each analyzed through 17 convolutional layers. The final outputs are then concatenated and processed further by 2 convolutional layers, then 2 fully connected layers and a final SoftMax layer which results in the scene classification (Figure 2). In the current study, we extracted the latent representations from the first 17 layers by concatenating the embeddings in the two branches along frequency, and the 2 subsequent convolutions layers and excluded the final fully connected layers following the same analysis procedure using for *M*_*event*_. The embeddings in each layer for each scene were then integrated over time resulting in a two-dimensional tensor with dimensions [*K*_*s*_, *F*_*s*_]^*l*^, where *K*_*s*_ represents the number of kernels, *F*_*s*_ represents frequency and *l* is the layer index.

#### Dimensionality Reduction.

Each network’s layer-wise latent representation for every scene was subsequently subjected to Principal Component Analysis (PCA; Wold et al., 1987). This step served the dual purpose of dimensionality reduction for further processing and the regulation of dominant dimensions, allowing a controlled and meaningful comparisons of activities across the two models. We opted for a reduced dimensionality of D = 136 following a selection process guided by two criteria: (1) capturing a minimum of 90% of the data variance across all embeddings, and (2) maintaining the same number of dimensions across layers and models for all global properties. This chosen value was employed in all subsequent analyses to evaluate the informative content of *each* layer and each model in relation to the behavioral scores. In a separate analysis, the embeddings from each layer of each model were concatenated together after the initial PCA (with D = 136). This tensor was then subjected to a second dimensionality reduction using PCA (with D = 136) to assess the global contribution of each model to the behavioral scores.

#### Regression Analysis.

Each of the eight empirically derived global properties was correlated with model representations using an independent linear regression. The variability in the human ratings for each property (averaged across subjects) across the 200 scenes was analyzed relative to: (a) the average embeddings for the two models (*M*_*setting*_ and *M*_*event*_) and (b) the embeddings per layer for each of the models. We analyzed all correlation trends using R_adj_^2^ to control for the effect of multiple predictors in reflecting the goodness of fit with behavioral data.

To compare the correlation trends across different layers for the two models, a curve fitting procedure was performed to the adjusted R_adj_^2^ using a bi-quadratic polynomial function, which used only statistically significant correlation values (*p*’s < 0.05). Since the number of layers in each model was not matched (14 for *M*_*event*_ versus 17 for *M*_*setting*_), the absolute label of the layers was converted to a relative scale between [0, 1] where 0 is the shallowest layer (*l* = 1) for both models and 1 is the deepest layer for both models.

## RESULTS

### Inter-Rater Reliability

To evaluate inter-rater reliability of ratings made by participants on each of the eight global property scales, intra-class correlation (ICC) coefficients and their 95% confidence intervals were calculated using IBM SPSS statistical software version 28 (see Table 2; IBM SPSS statistical software (Version 28)). We used a two-way random effects model based on average ratings to assess consistency across participants. The ICCs for all global property scales were statistically significant (all *p*-values < .001) and ranged from good to excellent (0.758–0.980; Koo & Li, 2016).

**Table 2.** Inter-rater reliability as measured by intra-class correlations (ICCs).

	ICC	95% CI		F value	F Test with True Value 0		
		Lower Bound	Upper Bound		*df*1	*df*2	*p* value
Sparseness	0.968	0.962	0.974	31.428	199	6965	< .001
Transience	0.944	0.933	0.955	17.919	199	6965	< .001
Season	0.758	0.707	0.804	4.135	199	6965	< .001
Navigability	0.787	0.742	0.827	4.667	199	6965	< .001
Open vs. Enclosed	0.925	0.909	0.939	13.288	199	6169	< .001
Outdoor vs. Indoor	0.977	0.972	0.981	43.402	199	6169	< .001
Natural vs. Human-Influenced	0.980	0.975	0.984	49.211	199	6169	< .001
Temperature	0.801	0.760	0.839	5.032	199	6169	< .001

*Note*. Results of ICC(2, k). Two-way random effects model, consistency definition, average measures. *df* = degrees of freedom.

### Accuracy of Global Property Judgments

**Figure 3.**
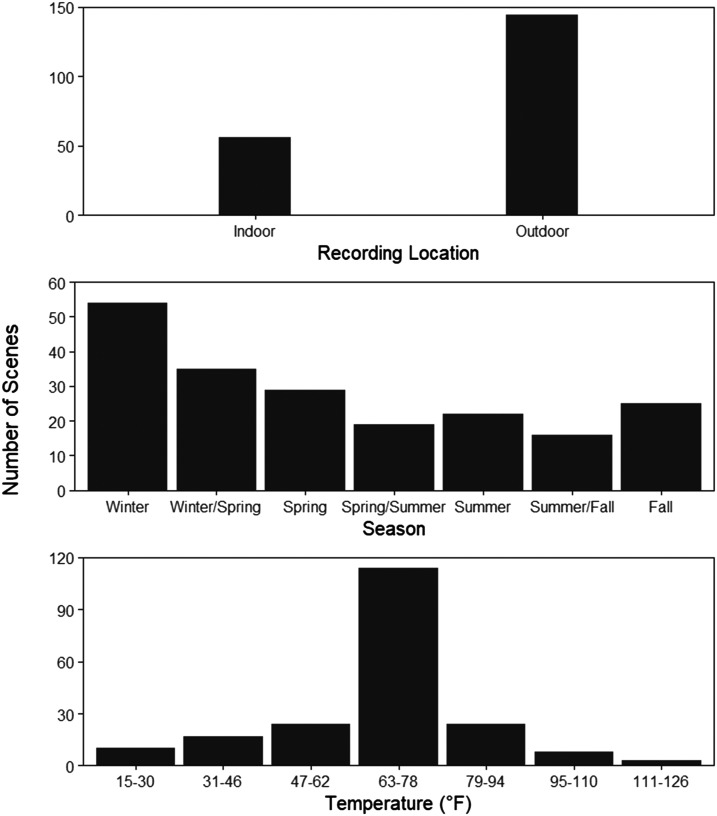
Distribution of Scenes. *Note*. Bar graphs representing the distribution of scenes in each of the following global properties: Outdoor vs. Indoor, Season, and Temperature.

**Figure 4.**
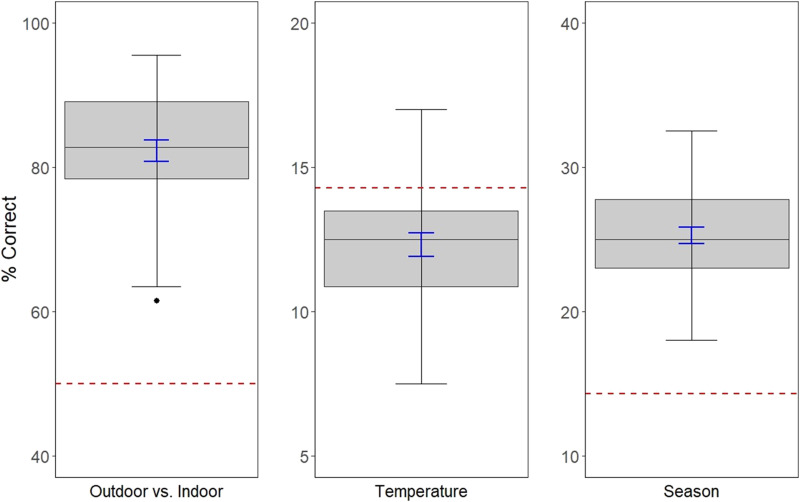
Accuracy of Global Property Judgments. *Note*. Boxplots showing the average percent correct scores for judgments made by participants on each of the following global properties: Outdoor vs. Indoor, Temperature, and Season. There is one outlier on the Outdoor vs. Indoor rating. The blue error bars represent standard error. The dashed red line represents the level of chance for each global property.

Percent correct was calculated to determine participant accuracy on judging the following global properties: Outdoor vs. Indoor, Temperature, and Season (see Figure 4). To calculate these scores, we referred to the metadata associated with each scene, which is included in our database (see Figure 3 for the distribution of scenes in each global property). For the Temperature property, we placed scenes into seven bins of 15 degrees each (e.g., bin 1 = 15–30°F, bin 2 = 31–46°F … bin 7 = 111–126°F) ranging from 15°F to 126°F. Next, we calculated each participant’s percent correct score based on their Temperature ratings to obtain an average score. Participants were correct 12.33% of the time on their ratings of Temperature, which is close to chance performance (14.29%).

Next, we categorized scenes according to the Season they were recorded in as it corresponds to the Season rating scale (e.g., Winter, Between Winter and Spring, etc., see Figure 1). We then calculated each participant’s percent correct score based on their Season ratings to obtain an average score, which revealed participants performed at 25.28%, which is above chance (14.29%). Finally, we categorized scenes as outdoor vs. indoor based on their recording location. We then dichotomized the outdoor vs. indoor scale and calculated percent correct based on participant ratings. Participants performed at 82.30%, which is well above chance (50%) on their ratings of Outdoor vs. Indoor. These results suggest participants can determine the season of a scene and whether it was recorded indoors or outdoors above chance, but not the temperature of a scene.

### Correlations Between Global Properties

Pearson correlations between average global property ratings of each scale are reported in Table 3. Overall, there are several moderate, strong, and very strong correlations between global property rating scales, which justifies their use in the exploratory factor analysis to determine the underlying factor structure of auditory scene perception.

**Table 3.** Summary of Means, Standard Deviation, and Correlations Among Average Global Property Ratings.

Variable	*M*	*SD*	1	2	3	4	5	6	7	8
1. Natural vs. Human-Influenced	4.55	1.61	—							
2. Open vs. Enclosed	3.05	0.95	.61***	—						
3. Outdoor vs. Indoor	3.15	1.55	.63***	.94***	—					
4. Temperature	3.81	0.47	.41***	.44***	.35***	—				
5. Navigability	4.45	0.54	−.25***	−.17*	−.12	−.35***	—			
6. Transience	3.62	0.73	.52***	.14*	.30***	−.03	.24***	—		
7. Season	4.17	0.56	.45***	.07	.17*	.03	.24***	.73***	—	
8. Sparseness	4.12	0.82	.35***	.05	.22**	−.07	.32***	.87***	.73***	—

*Note*. Correlations between average global property ratings for all auditory scenes (n = 200). * *p* < .05, ** *p* < .01, *** *p* < .001.

### Exploratory Factor Analyses

To evaluate the dimensionality of scene perception, we submitted the average global property ratings of each scale and all 35 acoustic measures to two separate exploratory factor analyses (EFA) using JASP statistical software version 16.1 (JASP Team, 2022).

#### Exploratory Factor Analysis: Global Properties.

**Figure 5.**
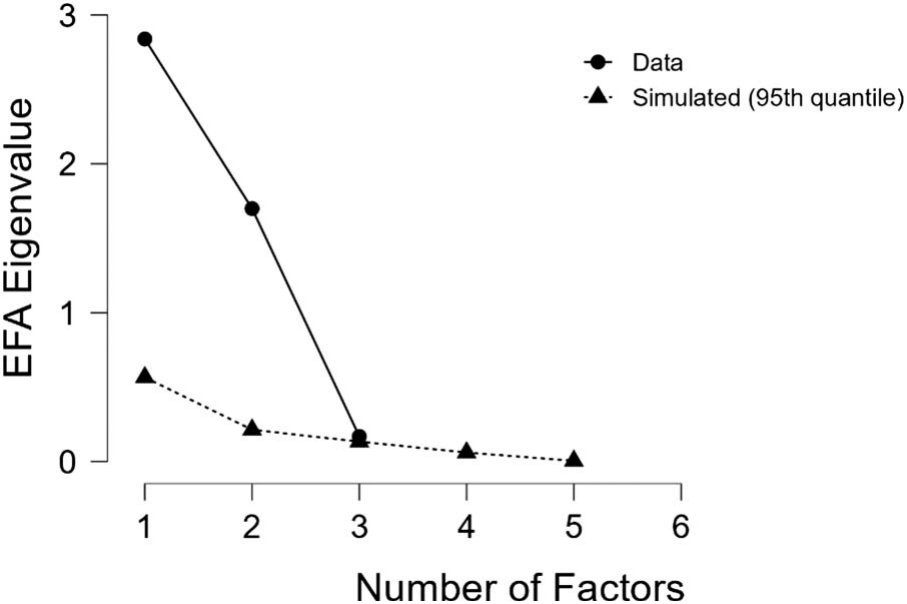
Scree plot of eigenvalues revealing a 2-factor model.

The average global property ratings of each scale (Naturalness, Openness, Sparseness, Navigability, Temperature, Outdoor vs. Indoor, Season, Transience) were entered into an EFA, with maximum likelihood factor extraction and Oblimin (oblique) rotation. The Kaiser-Meyer- Olkin test revealed sufficient sampling adequacy for the EFA, KMO = 0.69. Bartlett’s test of sphericity indicated the correlation structure of the variables was adequate for the EFA as well, *χ*^2^ (28) = 1253.84, *p* < .001. Upon visual inspection of the scree plot as well as a parallel analysis (see Figure 5), a two-factor solution was revealed and accounts for 64.0 % of the variance in the data. Table 4 displays the variables and factor loadings, with loadings less than |.40| excluded for clarity.

**Table 4.** Factor loadings of global property scales.

Factor Loadings		
	Factor 1	Factor 2
Transience	0.94	
Sparseness	0.93	
Season	0.78	
Navigability	0.37	
Open vs. Enclosed		1.01
Outdoor vs. Indoor		0.93
Natural vs. Human-Influenced		0.58
Temperature		0.46

*Note*. Extraction method: maximum likelihood; Rotation method: Oblimin (oblique).

##### Factor 1.

After rotation, Factor 1 consisted of four variables that accounted for 33% of the variance in the model. The global property variables which loaded onto this factor were Transience (0.94), Sparseness (0.93), Season (0.78). Although Navigability (0.37) also loaded onto this factor, its interpretation should be made with caution as its loading is below the |.40| threshold.

##### Factor 2.

Factor 2 consisted of four variables that accounted for 31% of the variance in model. The global property variables which loaded onto this factor were Openness (1.01), Outdoor vs. Indoor (0.93), Natural vs. Human-Influenced (0.58), and Temperature (0.46).

#### Exploratory Factor Analysis: Acoustic Variables.

All 35 acoustic measures (envelope-based intensity and rhythm measures, autocorrelation pitch statistics, correlogram-based pitch measures, moments of the spectrum, RMS energy in octave-wide frequency bands, spectral shift in time measures, modulation spectrum statistics) were submitted to a separate factor analysis using maximum likelihood factor extraction and Oblimin (oblique) rotation. Table 5 displays the variables and factor loadings for the rotated factors for the final model, with loadings less than |.40| excluded for clarity.

**Table 5.** Factor loadings of acoustic variables.

Factor Loadings							
	Factor						
	1	2	3	4	5	6	7
RMS in band *f*_*c*_ = 8000 Hz	0.89						
Moments of the Spectrum (Centroid)	0.87						
Moments of the Spectrum (SD)	0.81						
Mean Pitch	0.61				0.41		
Spectral Velocity (SD)		1.00					
Spectral Velocity (Maximum)		0.90					
Spectral Velocity (Mean)		0.86					
Modulation Statistics (Max Peak)		0.49					
Moments of the Spectrum (Skew)			−0.91				
Moments of the Spectrum (Kurtosis)			−0.86				
Spectral Flux (Mean)			−0.43		0.45		
Mean Peak in Autocorrelation				1.02			
SD of Peaks in Autocorrelation				0.98			
Maximum Peak in Autocorrelation					0.62		
Maximum Pitch Salience					0.62		
Mean Pitch Salience					0.58		
Spectral Flux (Maximum Peak)					0.48		
RMS in band *f*_*c*_ = 250 Hz					−0.43		
Pause-Corrected RMS Amplitude						0.97	
Overall RMS Amplitude						0.96	
Total Number of Bursts							0.90
Moments of the Spectrum (# of Peaks)							0.86

*Note*. Extraction method: maximum likelihood; Rotation method: Geomin (oblique); Loadings less than |.40| are not displayed.

**Figure 6.**
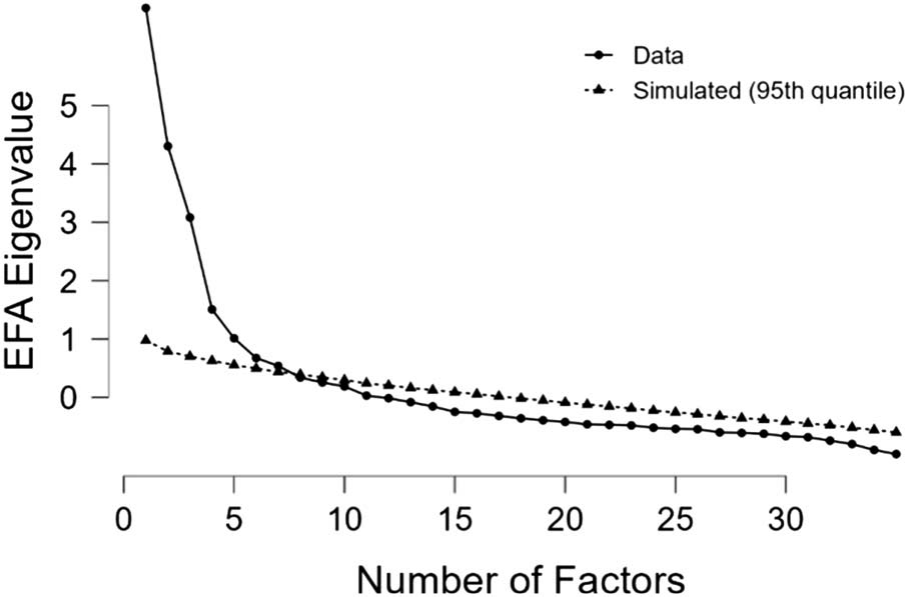
Scree plot of eigenvalues revealing a 7-factor model.

The Kaiser-Meyer-Olkin (KMO) test revealed sufficient sampling adequacy for the final EFA analysis, KMO = 0.71. Bartlett’s test of sphericity indicated the correlation structure of the variables was adequate for EFA as well, *χ*^2^ (595) = 7490.33, *p* < .001. Upon visual inspection of the scree plot as well as a parallel analysis (see Figure 6), a seven-factor solution was revealed and accounts for 57% of the variance in the data.

##### Factor 1.

After rotation, Factor 1 consisted of four variables that accounted for 10% of the variance in the model. One of the RMS energy measures of octave-wide frequency bands centered at 8000 Hz (0.89), two Moments of the Spectrum measures, the centroid (0.87) and standard deviation (0.81), and the mean pitch (0.61) all loaded onto this factor.

##### Factor 2.

Factor 2 consisted of four variables which accounted for 10% of the variance in the model. Three Moments of the Spectrum measures, the standard deviation (1.00), maximum (0.90), and mean (0.86), as well as one modulation statistics measure, the maximum peak (0.49) loaded onto this factor.

##### Factor 3.

Factor 3 consisted of three variables which accounted for 8% of the variance in the model. All these variables loaded negatively onto the factor, and they include two Moments of the Spectrum measures, the skew (−0.91) and kurtosis (−0.86), and the mean Spectral Flux (−0.43).

##### Factor 4.

Factor 4 consisted of two variables which accounted for 8% of the variance in the model. Both variables were measures of the autocorrelation, the mean peak (1.02) and standard deviation of peaks (0.98).

##### Factor 5.

Factor 5 consisted of seven variables which accounted for 8% of the variance in the model. Two variables also loaded onto other factors; the mean pitch (0.41) also loaded onto Factor 1, and the spectral flux mean (0.45) also loaded onto Factor 3. In addition, the maximum peak of the autocorrelation (0.62), maximum pitch salience (0.62), mean pitch salience (0.58), and the maximum peak of spectral flux (0.48) loaded onto this factor as well. One measure of RMS energy in octave-wide frequency bands centered at 250 Hz loaded negatively on this factor (−0.43).

##### Factor 6.

Factor 6 consisted of two variables which accounted for 7% of the variance in the model. Both variables were measures of RMS amplitude: the pause-corrected RMS (0.97) and overall RMS (0.96).

##### Factor 7.

Factor 7 consisted of two variables which accounted for 6% of the variance in the model. Both variables were measures of the envelope: burst duration/total duration (0.90) and number of bursts (0.86).

### Multiple Linear Regression Analyses

Next, eight multiple linear regression analyses were calculated to predict average ratings on each global property scale based on the acoustic measures. Overall, each global property rating scale was significantly predicted by at least one acoustic variable (see Table 6).

**Table 6.** Significant acoustic predictors for each global property rating scale.

Variable	*β*	R^2^
Natural vs. Human-Influenced		0.57
Pause-Corrected/Overall RMS Amplitude	0.16	
Pitch (Mean)	−0.44	
Pitch (SD)	−0.26	
Spectral Flux (SD)	−0.25	
Autocorrelation (Range of Peaks)	−0.14	
RMS in band *f*_c_ = 500 Hz	0.22	
Openness		0.57
Moments of the Spectrum (Centroid)	0.39	
Pause-Corrected RMS Amplitude	5.96	
Overall RMS Amplitude	−6.05	
Pitch (Mean)	−0.50	
Pitch (SD)	−0.20	
Spectral Flux (SD)	−0.30	
Autocorrelation (Range of Peaks)	−0.23	
RMS in band *f*_*c*_ = 250 Hz	0.18	
RMS in band *f*_*c*_ = 1000 Hz	−0.18	
Outdoor vs. Indoor		0.58
Moments of the Spectrum (Centroid)	0.35	
Pause-Corrected RMS Amplitude	4.89	
Overall RMS Amplitude	−4.94	
Pitch (Mean)	−0.57	
Pitch (SD)	−0.26	
Pitch Salience (Mean)	0.32	
Spectral Flux (SD)	−0.37	
Spectral Flux (Max Peak)	0.2	
Autocorrelation (Max Peak)	−0.23	
Autocorrelation (Range of Peaks)	−0.17	
RMS in band *f*_*c*_ = 1000 Hz	−0.14	
RMS in band *f*_*c*_ = 2000 Hz	0.14	
Temperature		0.33
Moments of the Spectrum (SD)	0.48	
Moments of the Spectrum (# of Peaks)	−0.34	
Pause-Corrected/Overall RMS Amplitude	3.68	
Spectral Velocity (Mean)	−0.6	
Spectral Velocity (SD)	0.86	
Navigability		0.41
Pause-Corrected/Overall RMS Amplitude	−0.17	
Season		0.56
Moments of the Spectrum (Skew)	−0.8	
Moments of the Spectrum (Kurtosis)	0.46	
Transience		0.78
Moments of the Spectrum (Skew)	−0.8	
Moments of the Spectrum (Kurtosis)	0.43	
Moments of the Spectrum (# of Peaks)	0.12	
Pitch (Mean)	−0.22	
Pitch Salience (Mean)	0.23	
RMS in band *f*_*c*_ = 500 Hz	0.19	
RMS in band *f*_*c*_ = 1000 Hz	0.2	
Sparseness		0.87
Moments of the Spectrum (Skew)	−0.97	
Moments of the Spectrum (Kurtosis)	0.51	
Moments of the Spectrum (# of Peaks)	0.09	
Pitch (Mean)	−0.15	
Pitch (Maximum)	0.13	
Pitch Salience (Mean)	0.27	
Spectral Flux (Mean)	−0.21	
Spectral Flux (SD)	−0.11	
RMS in band *f_c_* = 500 Hz	0.1	
RMS in band *f*_*c*_ = 1000 Hz	0.12	

#### Natural vs. Human-Influenced Regression.

The first regression was calculated to predict ratings on the Natural vs. Human-Influenced scale based on all 35 acoustic variables and was statistically significant, R^2^ = 0.57, R_adj_^2^ = 0.47, *F*(35, 162) = 6.09, *p* < .001. The significant acoustic predictors were pause- corrected/overall RMS amplitude (*β* = 0.16, *p* < .05), mean pitch (*β* = −0.44, *p* < .001), standard deviation of pitch (*β* = −0.26, *p* < .05), RMS energy in octave-wide frequency bands centered at 500 Hz (*β* = 0.22, *p* < .05), the spectral flux standard deviation (*β* = −0.25, *p* < .05), and the range of peaks in the autocorrelation (*β* = −0.14, *p* = .05).

#### Open vs. Enclosed Regression.

The regression predicting ratings on the Open vs. Enclosed scale based on all acoustic variables was statistically significant, R^2^ = 0.57, R_adj_^2^ = 0.48, *F*(35, 162) = 6.21, *p* < .001. The significant acoustic predictors were the Moments of the Spectrum centroid (*β* = 0.39, *p* < .05), pause-corrected RMS amplitude (*β* = 5.96, *p* < .05), overall RMS amplitude (*β* = −6.05, *p* < .05), mean pitch (*β* = −0.50, *p* < .001), standard deviation of pitch (*β* = −0.20, *p* < .05), spectral flux standard deviation (*β* = −0.30, *p* < .05), range of peaks in the autocorrelation (*β* = −0.23, *p* < .05), RMS energy In octave-wide frequency bands centered at 250 Hz (*β* = 0.18, *p* < .05), and 1000 Hz (*β* = −0.18, *p* < .05).

#### Outdoor vs. Indoor Regression.

The regression predicting ratings on the Outdoor vs. Indoor scale based on all acoustic variables was statistically significant, R^2^ = 0.58, R_adj_^2^ = 0.49, *F*(35, 162) = 6.33, *p* < .001. The significant acoustic predictors were the Moments of the Spectrum centroid (*β* = 0.35, *p* < .05), pause-corrected RMS amplitude (*β* = 4.89, *p* < .05), overall RMS amplitude (*β* = −4.94, *p* < .05), mean pitch (*β* = −0.57, *p* < .001) and standard deviation of pitch (*β* = −0.26, *p* < .05), mean pitch salience (*β* = 0.32, *p* < .05), spectral flux standard deviation (*β* = −0.37, *p* < .001), maximum peak in spectral flux (*β* = 0.20, *p* = .05), maximum peak in the autocorrelation (*β* = −0.23, *p* < .05), the range of peaks in the autocorrelation (*β* = −0.17, *p* < .05), RMS energy in octave-wide frequency bands centered at 1000 Hz (*β* = −0.14, *p* = .05) and 2000 Hz (*β* = 0.13, *p* = .05).

#### Temperature Regression.

A regression was calculated to predict ratings on the Temperature scale based on all acoustic variables and was statistically significant, R^2^ = 0.33, R_adj_^2^ = 0.19, *F*(35, 162) = 2.32, *p* < .001. The significant predictors were the Moments of the Spectrum standard deviation (*β* = 0.48, *p* < .05) and number of peaks (*β* = −0.34, *p* < .001), pause-corrected/overall RMS amplitude (*β* = 3.68, *p* < .05), spectral velocity mean (*β* = −0.60, *p* < .05), and spectral velocity standard deviation (*β* = 0.86, *p* < .05).

#### Navigability Regression.

The regression predicting ratings on the Navigability scale based on all acoustic variables was statistically significant, R^2^ = 0.41, R_adj_^2^ = 0.28, *F*(35, 162) = 3.18, *p* < .001. The only significant predictor was pause-corrected/overall RMS amplitude (*β* = −0.17, *p* < .05).

#### Season Regression.

A regression was calculated to predict ratings on the Season scale based on all acoustic variables and was statistically significant, R^2^ = 0.56, R_adj_^2^ = 0.47, *F*(35, 162) = 5.99, *p* < .001. The significant predictors were the Moments of the Spectrum skew (*β* = −0.80, *p* < .05) and kurtosis (*β* = 0.46, *p* < .05), as well as RMS energy in octave-wide frequency bands centered at 1000 Hz (*β* = 0.15, *p* < .05).

#### Transience Regression.

A regression was calculated to predict ratings on the Transience scale based on all acoustic variables and was statistically significant, R^2^ = 0.78, R_adj_^2^ = 0.73, *F*(35, 162) = 16.28, *p* < .001. The significant predictors were the Moments of the Spectrum skew (*β* = −0.80, *p* < .001), kurtosis (*β* = 0.43, *p* < .05), and number of peaks (*β* = 0.12, *p* < .05), mean pitch (*β* = −0.22, *p* < .05), mean pitch salience (*β* = 0.23, *p* < .05), as well as RMS energy in octave-wide frequency bands centered at 500 Hz (*β* = 0.19, *p* < .001) and 1000 Hz (*β* = 0.20, *p* < .001).

#### Sparseness Regression.

A regression was calculated to predict ratings on the Sparseness scale based on all acoustic variables and was statistically significant, R^2^ = 0.87, R_adj_^2^ = 0.84, *F*(35, 162) = 31.51, *p* < .001. The significant predictors were the Moments of the Spectrum skew (*β* = −0.97, *p* < .001), kurtosis (*β* = 0.51, *p* < .001), and number of peaks (*β* = 0.09, *p* < .05), mean pitch (*β* = −0.15, *p* < .05), maximum pitch (*β* = 0.13, *p* < .05), mean pitch salience (*β* = 0.27, *p* < .001), spectral flux mean (*β* = −0.21, *p* < .05), spectral flux standard deviation (*β* = −0.11, *p* < .05), as well as RMS energy in octave-wide frequency bands centered at 500 Hz (*β* = 0.10, *p* < .001) and 1000 Hz (*β* = 0.12, *p* < .001).

### Computational Modeling Results

**Figure 7.**
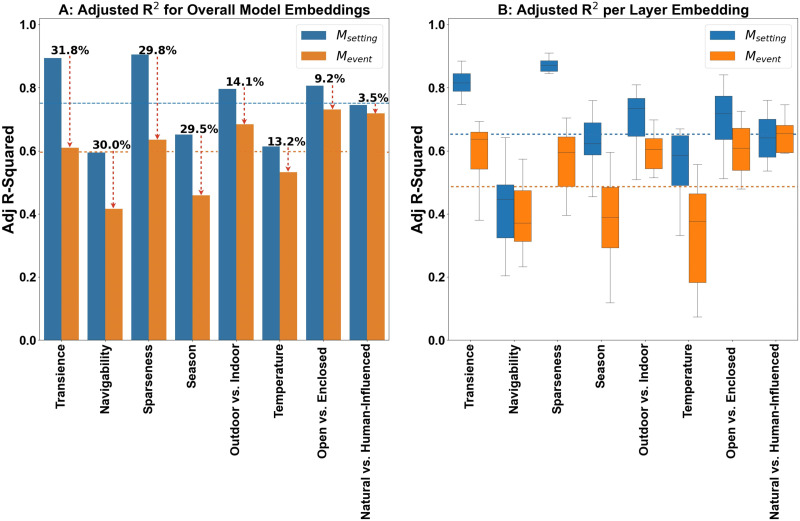
*Note*. (A) The bar graph depicts R_adj_^2^ values for each model across *aggregate* embeddings, with individual bars representing the correlation strength for each global property. The average adjusted R^2^ value for aggregate model embeddings across all global properties is shown as horizontal dashed lines. (B) R_adj_^2^ distributions for the *M*_*setting*_ and *M*_*event*_ models quantified for *each layer* of each model per global property. The average R_adj_^2^ value for layer wise model embeddings across all global properties is shown as horizontal dashed line.

Overall, when evaluating the predictive power of aggregate embeddings from the setting versus event models, adjusted R^2^ values are consistently higher for *M*_*setting*_ across all global properties (*M*_*setting*_ average aggregate R_adj_^2^ = 0.75, *M*_*event*_ average aggregate R_adj_^2^ = 0.59). Figure 7A illustrates the adjusted R^2^ values for aggregate representations from each model for individual global properties reported empirically. The regression values vary greatly across global properties for both models with explainable variance exceeding 80% for global properties like Transience (R_adj_^2^ = 0.89, *p* < .001) and Sparseness (R_adj_^2^ = 0.90, *p* < .001) for the *M*_*setting*_ model and as low as close to 40% for properties like Navigability (R_adj_^2^ = 0.41, *p* < .001) and Season (R_adj_^2^ = 0.45, *p* < .001) for the *M*_*event*_ model.

An important element that stands out from this analysis is the *relative* improvement in explanatory power for the *M*_*setting*_ model relative to the *M*_*event*_ model across all global properties. The results reveal that high difference in explanatory power for properties like Transience (ΔR_adj_^2^ = 31.8%), Navigability (ΔR_adj_^2^ = 30.0%), Sparseness (ΔR_adj_^2^ = 29.8%), and Season (ΔR_adj_^2^ = 29.5%). This difference in explanatory power across both models is slightly decreased for the global properties of Outdoor vs. Indoor (ΔR_adj_^2^ = 14.1%), Open vs. Enclosed (ΔR_adj_^2^ = 13.2%), and Temperature (ΔR_adj_^2^ = 9.2%), and even more severely reduced for the Natural vs. Human-Influenced (ΔR_adj_^2^ = 3.5%) global property. These differences may underlie contrasting representations in both models that can predict variability in human ratings of different properties across scenes and further support the divergence in factor loadings between properties like Transience, Navigability, Sparseness, and Season, as well as the other 4 properties (Outdoor vs. Indoor, Open vs. Enclosed, Temperature, Natural vs. Human-Influenced), which is in line with effects observed in the EFA reported in Table 4.

Looking closely at the variability of model embeddings across individual layers, Figure 7B reveals that—on average—layer-wise embeddings of the *M*_*setting*_ model are higher than those from the *M*_*event*_ model (*M*_*setting*_ layer-wise average R_adj_^2^ = 0.65, *M*_*event*_ average layer-wise R_adj_^2^ = 0.48). Nevertheless, the goodness of fit of individual layers to the behavioral ratings varies greatly across global properties. On the one hand, we note that global properties like Transience and Sparseness have a tight variance of for adjusted R^2^ (*M*_*setting*_ standard deviation across layers: Transience, *σ*_R^2^adj_ = 0.07, Sparseness, *σ*_R^2^adj_ = 0.06). This variance visibly increases for properties such as Navigability (*σ*_R^2^adj_ = 0.14), Open vs. Enclosed (*σ*_R^2^adj_ = 0.11), and Temperature (*σ*_R^2^adj_ = 0.11). On the other hand, the variability of regression fits across layers is generally higher for the *M*_*event*_ model. Properties such as Natural vs. Human-Influenced (*σ*_R^2^adj_ = 0.13) and Outdoor vs. Indoor (*σ*_R^2^adj_ = 0.14) reveal the lowest variability, while properties such as Navigability (*σ*_R^2^adj_ = 0.17) and Temperature (*σ*_R^2^adj_ = 0.16) have much higher variability across layers.

**Figure 8.**
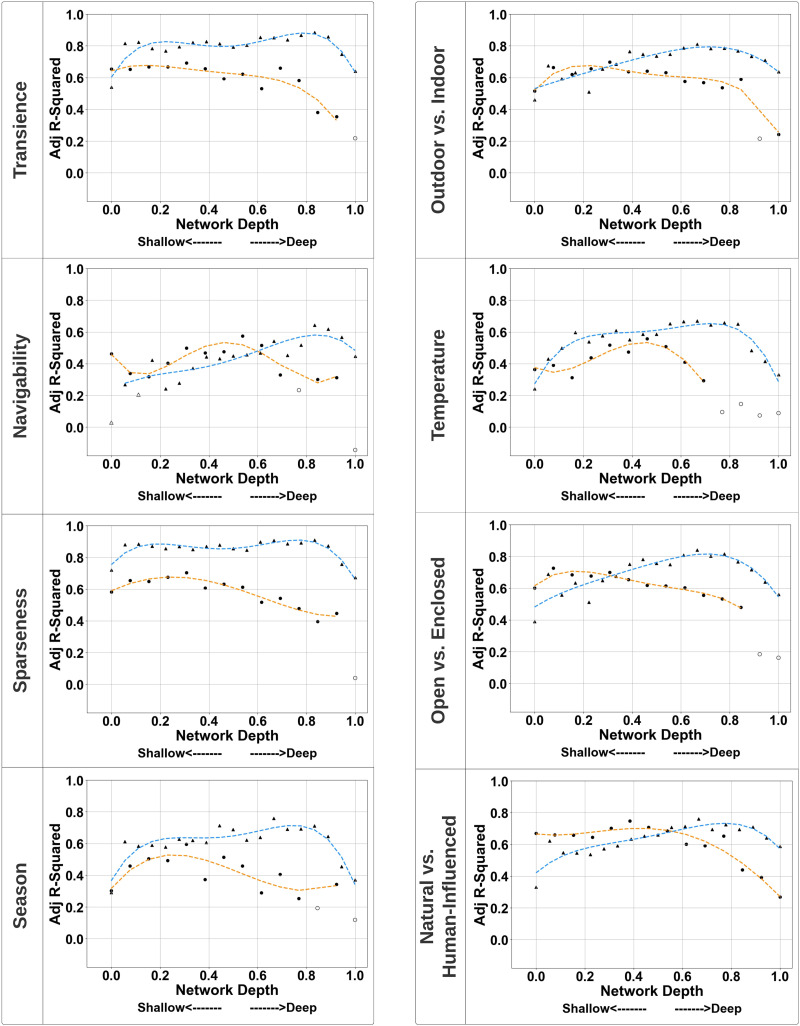
*Note*. Correlation between global properties and neural network hidden representations across all layers for *M*_*event*_ (in orange) and *M*_*setting*_ (in blue) networks. The y-axis signifies the adjusted R^2^ values, and the x-axis showcases the normalized relative depth from the shallowest (0) to the deepest layer (1).

Figure 8 looks closely at the trend of correlations for each global property across the layers of both models and again underscores the general advantage of the setting model especially in explaining variability across global properties like Transience, Sparseness, and Season. Furthermore, we note differences in predictive power across the network layers for both models, with the *M*_*setting*_ model revealing correlation peak at deeper points of the network relative to the *M*_*event*_ model across global properties. The curve fit for *M*_*setting*_ model shows a peak corresponding to relative depth of 0.77 for Transience, 0.83 for Navigability, 0.77 for Sparseness, 0.72 for Season, 0.72 for Outdoor vs. Indoor, 0.72 for Temperature, 0.72 for Open vs. Enclosed, and 0.77 for Natural vs. Human-Influenced. Conversely, the *M*_*event*_ model appears to lose predictive power in the deeper layers of the model across all properties and shows a peak corresponding to relative depth of 0.15 for Transience, 0.46 for Navigability, 0.23 for Sparseness, 0.23 for Season, 0.23 for Outdoor vs. Indoor, 0.46 for Temperature, 0.15 for Open vs. Enclosed, and 0.46 for Natural vs. Human-Influenced. Here, a relative depth of 0 represents the shallowest layer and 1 represents the deepest layer. The normalization facilitates a direct comparison between the two models, which have differing layer counts (see Methods). These peaks are likely driven by great variability in analysis time constants and accumulation of nonlinearities across the model layers from shallower to deeper processing stages (Figure 2).

## GENERAL DISCUSSION

Here, we investigated the contributions of high-level global information and low-level acoustic features to auditory scene perception. Participants listened to complex, real-world auditory scenes and made judgments on a series of global properties (Sparseness, Transience, Season, Navigability, Open vs. Enclosed, Outdoor vs. Indoor, Natural vs. Human-Influenced, Temperature). We found high between-participant consistency on ratings of all eight global property rating scales (indicated by the significant intra-class correlations). This particular result provides preliminary evidence for the ability to perceive auditory scenes from a global perspective, which is consistent with findings in the visual domain (Greene & Oliva, 2009a). A variety of acoustic measures were useful in predicting each of the global property ratings, though some of the acoustic-global relationships were non-linear. Additionally, the results of the computational modeling analysis provide preliminary evidence that individual sound events within a scene may not be the primary drivers of global judgments. This supports the notion that the scene’s overarching acoustic structure and its contextual setting largely influence the perceptual judgments of these global ratings.

These results are consistent with the hypothesis that global scene properties serve as high-level dimensions to inform a scene’s layout, function, and constancy, allowing observers to rapidly understand the scene without needing to identify individual objects that are present (Greene & Oliva, 2009a, 2009b, 2010; Ross & Oliva, 2010). Using both behavioral and computational methods, prior studies have demonstrated that observers can more quickly and accurately categorize visual scenes into a global category (e.g., an open environment) than a basic level category (e.g., a waterfall; Greene & Oliva, 2009a), use global information to perform basic-level categorization tasks (Greene & Oliva, 2009b), and adapt to global properties of visual scenes (Greene & Oliva, 2010). Additional electrophysiological studies have indicated the P2 event related potential (ERP) as a neural marker for global scene properties (Harel et al., 2016) and have revealed that global scene information is extracted in early ERP components (P1, N1, and P2; Hansen et al., 2018). The mounting evidence for the use of global scene properties in the visual domain provides a promising foundation for future behavioral, electrophysiological, neuroimaging, and computational studies in the auditory domain. Although our study only measured eight global properties, many more may be uncovered and investigated by future research to provide a well-rounded understanding of how people interpret complex auditory scenes. For example, future studies can evaluate how well people can identify the setting of a scene compared to the objects within it. Additionally, it would be useful to measure how quickly and in what order global scene judgments are made (e.g., is openness perceived prior to temperature?) and whether observers can adapt to these properties.

### Dimensionality of Auditory Scene Perception

The results of our study indicate a high amount of dimensionality reduction along the auditory pathway when we listen to auditory scenes. Dimensionality reduction has been demonstrated in the perception of environmental sounds (Gygi et al., 2007), timbre (Grey, 1977), musical tonality (Krumhansl, 1979; Shepard, 1982; Toiviainen et al., 1995), and rhythm (Desain & Honing, 2003; Jacoby & McDermott, 2017), suggesting this is a common feature of auditory processing. The multiple linear regressions calculated to predict performance on the global property rating scales based on the acoustic properties of each scene revealed each rating scale was significantly predicted by at least one acoustic variable. This finding, along with the difference in the number of reduced factors in the exploratory factor analyses (2 global property factors vs. 7 acoustic factors), suggest global variables may be processed at a higher level of the auditory pathway where acoustic features have been abstracted out of or have nonlinear relationships with global variables. Although the EFA on the global properties yielded a factor structure which mirrors the judgments made by our groups of participants (i.e., the global properties that loaded onto Factor 1 correspond to judgments made by Groups 1–4, and those that loaded onto Factor 2 correspond to judgements made by Groups 5–8), the results of our modeling analysis suggest this is not purely an artifact of our study design. When we compared the explanatory power for all global properties between the setting- and object-based models (Figure 8), we found a high difference in power for the global properties that loaded onto Factor 1 (Navigability, Transience, Sparseness, and Season) while a much smaller difference was found for properties that loaded onto Factor 2 (Outdoor vs. Indoor, Open vs. Enclosed, Temperature, and Natural vs. Human-Influenced). These differences in explanatory power across models suggest there are underlying differences in the representations in both models, further supporting the results of the EFA. The transformation of low-level acoustic information into high-level global representations of auditory scenes could occur similarly to processing along the ventral visual stream, where low- level information about visual objects (e.g., an object’s geometric shape, position in space, etc.) culminates into high level representations of visual objects which allow for object recognition (DiCarlo et al., 2012). Additionally, common neural population codes have been shown to have highly nonlinear relationships, suggesting nonlinear transformation is a common feature of sensory processing (De & Chaudhuri, 2023). The results of the modeling analysis provide additional evidence for this conclusion, revealing the best predictive power across all global properties is achieved after several transformations of the time-frequency input, which ultimately highlights the critical role of information abstraction that underlies the processing hierarchy in these deep models (Kell & McDermott, 2019; Yamins & DiCarlo, 2016). A similar abstraction is a hallmark of sensory pathways, and particularly at the level of auditory cortex whereby hierarchical mappings and functional specializations appear to facilitate various complex auditory tasks (Bizley & Cohen, 2013; de Heer et al., 2017; Kumar et al., 2007; Okada et al., 2010).

Investigating responses to auditory scenes along the auditory pathway will be essential to our understanding of how the auditory system integrates low-level acoustic features of auditory scenes into high-level global representations of scenes. The auditory system functions hierarchically, showing tuning specificity for simple stimuli and acoustic features, such as pitch (Bendor & Wang, 2005; Norman-Haignere et al., 2013; Patterson et al., 2002), frequency (Da Costa et al., 2011; Humphries et al., 2010), spatial cues (Higgins et al., 2017; Rauschecker & Tian, 2000; Stecker et al., 2005), and spectral and temporal modulations (Barton et al., 2012; Chi et al., 2005; Santoro et al., 2014; Schönwiesner & Zatorre, 2009) in primary auditory areas as well as tuning specificity for more complex stimuli, such as noise bursts (Kaas & Hackett, 2000), vocalizations (Belin et al., 2000; Petkov et al., 2008; Rauschecker & Tian, 2000), speech (Mesgarani et al., 2014; Norman-Haignere et al., 2015; Overath et al., 2015; Scott et al., 2000), song (Norman-Haignere et al., 2022), and music (Boebinger et al., 2021) in non-primary auditory areas. The increase in response complexity along the auditory pathway suggests sound features are abstracted from combinations of more simple responses, such as the acoustic features of sounds, which parallels findings in the visual system (Carandini et al., 1999; Cumming & DeAngelis, 2001; De Valois & De Valois, 1980; DiCarlo et al., 2012; Gegenfurtner & Kiper, 2003; Horwitz & Hass, 2012; Hubel & Wiesel, 1962; Movshon et al., 1978; Tootell et al., 1988, 1998).

### Neural Pathways for Scene Processing

The existence of global properties is supported by behavioral, computational (Greene & Oliva, 2009a, 2009b, 2010; Ross & Oliva, 2010), and neural (Hansen et al., 2018; Harel et al., 2016) studies in the visual domain as well as the results of our behavioral study. This raises important questions regarding the neural pathways allowing for global processing of auditory scenes.

The computations underlying auditory perception are suggested to occur along two parallel processing streams analogous to the dorsal and ventral streams in the visual domain (Goodale & Milner, 1992; Milner & Goodale, 2006; Mishkin et al., 1983), allowing us to identify where a sound is coming from and also identify what we are listening to (Alain et al., 2001; Griffiths, 2008; Lomber & Malhotra, 2008; Rauschecker, 1998; Rauschecker & Tian, 2000). The dorsal auditory stream affords the ability to localize auditory stimuli in space (Rauschecker, 2012; Rauschecker & Scott, 2009) and map sounds onto motor-based representations involved in speech production (Hickok & Poeppel, 2004), while the ventral auditory stream affords identification and semantic processing of auditory stimuli (including speech).

The ventral auditory stream originates in the core auditory fields A1 and R, continues to the anterolateral and middle lateral belt regions, and terminates in the ventrolateral prefrontal cortex (vlPFC; Kaas & Hackett, 2000; Rauschecker & Tian, 2000). Neurons in the core prefer lower-level sound features such as frequency and intensity, while neurons in the anterolateral belt respond to vocalizations, frequency-modulated sweeps, and band-passed noise (Chang et al., 2010; Rauschecker et al., 1995; Rauschecker & Tian, 2000, 2004; Tian & Rauschecker, 2004; Tian et al., 2001).

Many neurophysiological and neuroimaging studies have identified cortical regions with selectivity for distinct aspects and categories of auditory input (e.g., voices, environmental sounds, music, etc.). Pitch is an important perceptual feature of many sounds, including speech, music, and environmental sounds, and it allows us to identify voices, segregate and organize sounds in complex auditory scenes, and convey musical structure and emotion. Pitch-selectivity has been demonstrated in anterolateral regions of A1 (Bendor & Wang, 2005, 2006, 2010; Norman-Haignere et al., 2013, 2015; Penagos et al., 2004; Patterson et al., 2002) and speech-selective regions have been localized further along the auditory pathway along the middle and anterior superior temporal sulcus (STS) and superior temporal gyrus (STG; Belin et al., 2000, 2002; Boebinger et al., 2021; Chang et al., 2010; Davis & Johnsrude, 2003; Mesgarani et al., 2014; Norman-Haignere et al., 2015, 2022; Pernet et al., 2015; Yi et al., 2019). Music-selective populations have been observed in areas anterior and posterior to A1, while speech-selective populations have been observed in areas lateral to A1, which suggests music and speech representations begin to diverge in non-primary auditory cortex and are potentially processed in partially distinct pathways along the ventral stream. A recent study identified a song-selective (i.e., music with singing) population co-located with music and speech selective regions (Norman-Haignere et al., 2022), suggesting multiple neural populations exist that are selective for particular aspects of music (e.g., singing), and further demonstrates the complexity of processing within the auditory cortex.

Selectivity for animal sounds, voices, human-made sounds, and tools have been localized to middle temporal gyrus (MTG), STS, and STG (Bethmann & Brechmann, 2014; Lee et al., 2009; Sharda & Singh, 2012; Zhang et al., 2015, 2020), and a recent human electrocorticography (ECoG) and fMRI study found selectivity for a variety of environmental sounds (e.g., whistling, telephone dial, wind chimes, car horn, splashing water, etc.) in posterior primary auditory cortex, which may suggest a third stream exists in the auditory cortex for environmental sound processing (Norman-Haignere et al., 2022). The processing of environmental sounds may not emerge until later along the ventral stream, potentially closer to or in the vlPFC, where processing may reflect post-sensory processes such as auditory attention, working memory, the meaning of sounds, and integration of multisensory information and aid in our perception of many categories of sound stimuli (Cohen et al., 2009; Lee et al., 2009; Ng et al., 2014; Plakke & Romanski, 2014; ; Poremba et al., 2003; Romanski et al., 2005; Russ, Ackelson, et al., 2008; Russ, Orr, & Cohen, 2008).

There is evidence of both object-selective and scene-selective regions (Epstein & Baker, 2019) along the ventral visual stream. Epstein and Kanwisher (1998) identified the parahippocampal place area (PPA), a region of the cortex which responds more strongly to pictures of scenes (e.g., houses) than objects (e.g., bodies, faces) during both active perception and mental imagery of visual scenes. More recent studies have highlighted the role of PPA in recognition of non-visual information as well, such as descriptions of famous places (Aziz-Zadeh et al., 2008) and audio descriptions of spatial information (Häusler et al., 2022). Although not located in the ventral visual stream, the medial place area (MPA) and occipital place area (OPA) have demonstrated roles related to visually guided navigation and map-based navigation, respectively (Epstein & Kanwisher, 1998; Dilks et al., 2013, 2022; Nakamura et al., 2000; O’Craven & Kanwisher, 2000). An additional fMRI study identified a series of distributed cortical networks which show tuning specific to various scene categories (e.g., navigation, social interaction, human activity, motion-energy, texture, non-human animals, civilization, natural environment), demonstrating the complexity of scene processing in the human cerebral cortex (Çelik et al., 2021).

Many object-specific areas have been identified as well; some areas respond most to faces (fusiform and occipital face areas; Haxby et al., 2000; Kanwisher et al., 1997), shapes (posterior fusiform and lateral occipital complex (LOC); Malach et al., 1995; Grill-Spector & Malach, 2004), or bodies (fusiform and extrastriate body area; Downing et al., 2001; Peelen & Downing, 2005). A replication of Dilks et al. (2013) provided further evidence of a double dissociation in scene and object processing in the OPA and LOC. Transcranial magnetic stimulation (TMS) delivered to OPA impaired the recognition of scenes while TMS delivered to LOC impaired recognition of objects (Wischnewski & Peelen, 2021). Scene and object processing has also been explored using intracranial electroencephalography (iEEG), which offers high anatomical and temporal resolution. Vlcek et al. (2020) collected iEEG data as epileptic patients viewed images containing both objects and scenes. Their results support the roles of the PPA and LOC in scene and object processing, respectively, as well as scene-selective areas MPA and OPA. This scene network was shown to extend to regions involved in processing scene novelty (anterior temporal lobe regions, including the hippocampus and parahippocampal gyrus). Additional object-selective areas were identified, including areas selective for tool use (intraparietal sulcus, supramarginal gyrus and middle temporal cortex; Vidal et al., 2010) and object recognition (inferior frontal gyrus and perirhinal cortex; Bar et al., 2001; Clarke & Tyler, 2014; Nakamura et al., 2000).

Future neuroimaging studies or invasive neurophysiological studies will be necessary to evaluate how complex auditory scenes are processed along auditory-specific pathways. An additional research avenue could investigate the potential relationship between the ventral visual stream and auditory scene processing; it is possible that visual areas may aid in the perception and representations of auditory scenes. Additionally, computational modeling studies could better our understanding of the neural and computational processes contributing to auditory scene processing. Most models of auditory scene analysis focus on the segregation of two auditory stimuli (e.g., tones, noise bursts, speech, foreground/background) into perceptual streams (Elhilali & Shamma, 2008; Krishnan et al., 2014; Ma, 2011), but these models are limited and do not explain how auditory objects and scenes are identified or understood. One model of the auditory system assessed the recognition and understanding of synthesized sound textures (i.e., temporally homogenous sounds such as a rainstorm or a choir of crickets; McDermott & Simoncelli, 2011); however, future studies are necessary to evaluate how auditory objects and scenes are processed from early to late stages in the auditory system.

### Limitations

There are potential limitations of this study. Participants were not provided with examples of scenes at low, medium, and high levels of each global property scale, similar to Greene and Oliva (2009a, 2009b). Including examples of scenes at either extreme of each global property could provide participants with a better understanding of the task and should thus be included in future studies. The lack of examples could have made the scales harder to interpret; however, we are confident participants used each scale consistently since our measures of inter-rater reliability showed high agreement (see Table 2). Although ratings of global properties showed high agreement, it is possible that participants could not accurately rate some scales, such as temperature. Future studies should investigate other global variables that can be perceived more accurately in the auditory domain. It is also important to note the rating scale for Season did not include an option for “Between Fall and Winter,” which could have affected the results obtained from this scale.

There are many factors that may have influenced the degree to which participants used the global properties of scenes and/or objects within the scenes to make their judgments. The scene duration (4 seconds) could have led participants to rely on objects and the overall setting of each scene rather than global properties to make their judgments. We were not confident participants would be able to extract as much information from shorter durations (e.g., 1 or 2 seconds) compared to a longer duration of 4 seconds. However, if this experiment were to be repeated in the future with shorter scene durations, it is possible that participants could make judgments based more on the global properties instead of individual objects within the scenes. Additionally, the inter-rater reliability scores and factor structure could differ from what is reported here if shorter scene durations were to be used.

Further, this study is correlational, and used a relatively small group of 200 scenes. In the future, a more powerful design could use a higher number of scenes that vary in setting and types of objects present within each scene. Finally, this study was conducted online; although we included a headphone check and attention check, we did not have control over distractions, or the quality of headphones used by participants.

### Conclusions

In summary, our results provide preliminary evidence for the ability to perceive auditory scenes from a global perspective. Additionally, the results of both the behavioral experiment and computational modeling analysis suggest a high degree of dimensionality reduction along the auditory pathway wherein global properties of scenes are processed at a high level and the acoustic features of scenes are processed at a low level. Examining the role of global properties as well as individual sound events in our perception of auditory scenes is essential to gain a finer understanding of the underlying processes which construct our representations of the auditory environment.

## ACKNOWLEDGMENTS

The authors wish to thank Dr. Jessica Nave-Blodgett, Chastity Balagtas, Jared Leslie, Sarah Flood, Lucy Pineda-Roman, Lan Nguyen, and Dr. Karli M. Nave for their assistance with collecting and editing auditory scenes for our database. They also thank Dr. Colleen M. Parks and Dr. Erin E. Hannon for their expertise and feedback on the methods and writing of this manuscript, as well as Rodica R. Constantine for her scientific insights, feedback, and general support of this project. Finally, the authors thank the reviewers whose valuable comments and suggestions helped to improve the quality of this manuscript.

## FUNDING INFORMATION

Margaret A. McMullin was supported by the Department of Defense through the National Defense Science & Engineering Graduate (NDSEG) Fellowship Program. This work was partially supported by ONR N00014-23-1-2050.

## AUTHOR CONTRIBUTIONS

M. A. M.: Conceptualization and design; Data collection; Formal analysis; Visualization; Writing – original draft; Writing – Review & editing. R. K.: Conceptualization and design; Formal Analysis; Visualization; Writing – original draft; Writing – Review & editing. N. C. H.: Data cleaning and analysis; Writing – Review & editing. B. G.: Data analysis; Writing – Review & editing. M. E.: Conceptualization and design; Formal Analysis; Supervision; Writing – original draft; Writing – Review & editing. J. S. S.: Conceptualization and design; Formal Analysis; Supervision; Writing – original draft; Writing – Review & editing.

## DATA AVAILABILITY STATEMENT

The data and stimuli are available at OSF: https://osf.io/zj4xe/.
